# Network based approach identifies *miR* *‐145‐3p* as a central regulatory hub associated to the progression from localized to metastatic medullary thyroid carcinoma

**DOI:** 10.1002/path.70116

**Published:** 2026-09-09

**Authors:** Anna Citarella, Manuela Petti, Zein Mersini Besharat, Caterina Alfano, Elena Splendiani, Sofia Trocchianesi, Sara Cassandro, Martina Chiacchiarini, Claudia Sabato, Francesca Gianno, Tanja Milena Autilio, Martina Brunetti, Giuseppina Catanzaro, Antonella Verrienti, Rossella Elisei, Raffaele Ciampi, Cristina Romei, Maria Grazia Castagna, Silvia Cantara, Vincenzo Marotta, Luciano Pezzullo, Lorenzo Farina, Cosimo Durante, Agnese Po, Elisabetta Ferretti

**Affiliations:** ^1^ Department of Life Science, Health, and Health Professions Link Campus University Rome Italy; ^2^ Department of Computer, Control and Management Engineering Sapienza University of Rome Rome Italy; ^3^ Department of Experimental Medicine Sapienza University of Rome Rome Italy; ^4^ Laboratory of Preclinical and Translational Research, Centro di Riferimento Oncologico della Basilicata, (IRCCS‐CROB) Rionero in Vulture (Potenza) Italy; ^5^ Department of Radiological Sciences, Oncology and Pathology Sapienza University of Rome Rome Italy; ^6^ Department of Translational and Precision Medicine Sapienza University of Rome Rome Italy; ^7^ Endocrine Unit, Department of Clinical and Experimental Medicine University of Pisa Pisa Italy; ^8^ Department of Medical, Surgical and Neurological Sciences University of Siena Siena Italy; ^9^ Department of Medicine, Surgery and Dentistry, ‘Scuola Medica Salernitana’ University of Salerno Baronissi Italy; ^10^ Clinica Sanatrix SPA – Napoli e Pineta Grande Hospital SPA ‐ Castel Volturno Caserta Italy

**Keywords:** medullary thyroid carcinoma, microRNAs, network analysis, metastatic progression, *miR‐145‐3p*, MAPK signaling

## Abstract

Medullary thyroid carcinoma (MTC) is a neuroendocrine tumor originating from calcitonin producing C‐cells and accounts for 1–5% of thyroid cancers. Total thyroidectomy is curative in localized disease (N0), whereas lymph node metastases (N1) are associated with poorer prognosis. However, the molecular mechanisms driving the metastatic shift remain poorly understood. This study aimed to identify miRNA features linked to metastatic spread in MTC, focusing on the transition from N0 to N1. Co‐expression networks were constructed for N0 and N1 tumors, and differential connectivity analysis was used to identify key miRNAs acting as regulatory hubs. Functional annotation of their target genes was performed using the Kyoto Encyclopedia of Genes and Genomes (KEGG), Gene Ontology (GO), and Reactome pathway analyses. Validation experiments were carried out in MTC cells to evaluate the effects of selected miRNAs on cell proliferation, survival, and MAPK pathway activation. Network analysis revealed distinct miRNA co‐expression patterns between N0 and N1 tumors. Differential network analysis highlighted *miR‐145‐3p* as a central regulatory hub, exhibiting 29 altered co‐expression changes and a marked loss of connectivity in N1. Target enrichment identified 59 validated genes, including key oncogenic drivers such as *MYC*, *PTEN*, *BCL2*, *PIK3CA*, *AKT1*, and *MAPK7*. In MTC cells, simultaneous inhibition of *miR‐145‐3p* together with its top co‐expressed miRNAs increased proliferation and survival, and enhanced ERK phosphorylation, indicating MAPK pathway activation and a shift toward a more aggressive phenotype. In conclusion, this study identifies a miRNA regulatory hub centered on *miR‐145‐3p* that is associated with metastatic progression and highlights the value of network‐based approaches in uncovering mechanisms of cancer dissemination. © 2026 The Author(s). *The Journal of Pathology* published by John Wiley & Sons Ltd on behalf of The Pathological Society of Great Britain and Ireland.

## Introduction

Medullary thyroid carcinoma (MTC) is a rare neuroendocrine tumor arising from parafollicular, calcitonin (Ctn)‐producing C‐cells. This tumor represents from 1% to 5% of all thyroid malignancies and can occur sporadically in 75–80% of cases or as part of hereditary syndromes in the remaining cases. The most common mutations associated with MTC occur in the Rearranged During Transfection (RET) proto‐oncogene, and in the RAS gene family [1]. MTC diagnosis relies on the identification of the thyroid nodule, evaluated through neck ultrasound and fine‐needle aspiration, along with high serum Ctn levels [1]. Surgery is the mainstay treatment for MTC; currently total thyroidectomy is often recommended because of the tumor's multifocal nature and the high risk‐related recurrence [2, 3]. This approach is generally curative for patients with localized MTC (stage I or stage II), who frequently have a good prognosis, with a 10‐year survival rate of 95.6%. Conversely, patients with distant metastases have a more severe prognosis, with a 10‐year survival rate of 40%; patients with spreading at local lymph nodes have an intermediate prognosis [1] (10‐year survival rate of 70%). In this context, nodal spreading represents the first step of the metastatic process, and it can happen in an early stage of the disease. Even though several molecular aspects have been elucidated in MTC biology, the mechanisms driving early dissemination are still unclear. A better understanding of the molecular events underlying the transition from N0 to N1 condition may lead to identification of the molecular key players involved in aggressiveness, therefore discovering new putative targets for therapeutic treatment. In this context, microRNAs (miRNAs) are small non‐coding RNAs that regulate gene expression at the post‐transcriptional level and are under intense investigation as biomarkers for diagnosis and prognosis and as regulators of biological processes in several pathologies, including cancer. In MTC, their pivotal role in regulating cancer development progression and as novel diagnostic biomarkers has already been demonstrated [4, 5, 6].

Advances in data analysis approaches have driven the development and implementation of novel tools, enabling researchers to achieve a more comprehensive molecular characterization. In this context, network analysis based on mathematical graph theory allows the study of a complex system (i.e. complex diseases such as cancer) that emerges from interactions between its individual components. Networks have been widely used in recent years to conduct in‐depth investigations by modeling different omics data [7], and differential network analysis has become an important tool for revealing the network rewiring between two cancer stages [8]. This study aimed to exploit network analysis to identify tissue miRNAs specific to early metastatic disease in MTC. To this aim, we analyzed the miRNA profiles of 28 primary MTC tissues belonging to two staging subgroups: no nodal metastasis (N0) compared with the presence of nodal metastasis (N1) and performed co‐expression network and differential network analysis to identify miRNAs with a potential role in early metastatic spread, and therefore signaling pathways involved in early spreading.

## Materials and methods

### Ethics approval and patient consent

Ethics approval for the MTC patient cohort of this study was obtained from the ethical committee of the Policlinic Umberto I – Sapienza University of Rome (protocol: OTC‐CBSS‐1114, ethical committee reference: 4940). All participants provided written informed consent in accordance with the Declaration of Helsinki (https://www.wma.net/policies‐post/wma‐declaration‐of‐helsinki/).

### Patient samples

Tumor samples from 28 patients with sporadic MTC who underwent total thyroidectomy and had not previously received treatment or had any previous or concurrent neoplasm were collected from the following centers: Sapienza University of Rome, Università degli Studi di Siena, Azienda Ospedaliero‐Universitaria Pisana, Pisa, and Istituto Nazionale Tumori IRCCS—Fondazione G. Pascale, Napoli, with the approval of the institutional review board (protocol: OTC‐CBSS‐1114, ethical committee reference: 4940). The pathologist of each center reviewed the samples and all clinic‐pathologic features are shown in supplementary material, Table S1A,B. Thirteen patients with MTC had localized disease (N0M0), and 15 had nodal metastasis (N1M0) at diagnosis following the American Joint Committee on Cancer (AJCC) classification. The follow‐up of surviving patients ranged from 30 months to over 10 years, with a median of 40 months.

### miRNA profiling

For formalin‐fixed, paraffin‐embedded (FFPE) MTC samples, RNA was extracted using Norgen Tissue Samples FFPE RNA Extraction Kit (Cat. 25300; Norgen Biotek Corp., Thorold, Ontario, Canada) from one 10 μm‐thick slice after the selection of the relevant portion of the tissue by the pathologist. For the fresh‐frozen (FF) MTC samples, RNA extraction was performed using TRIzol (Cat. 15596026; Life Technologies, Thermo Fisher Scientific, Waltham, MA, USA) following the manufacturer's protocol, followed by cleanup with the miRNeasy Kit (Cat. 217084; Qiagen GmbH, Hilden, Germany). miRNA retro‐transcription was performed from 10 ng of total RNA using TaqMan Advanced miRNA cDNA Synthesis Kit (Cat. A28007; Life Technologies). miRNA expression profiling was performed using TaqMan Low Density Array (TLDA) microfluidic cards (A31805; Life Technologies) following the manufacturer's protocol.

### miRNA expression analysis

Raw data from miRNA profiling were processed using R packages HTqPCR, corrplot, ggplot2, ggrepel, and heatmap3. Data were cleaned, filtered, and normalized, and expression analysis was performed using corrplot, ggplot2, ggrepel, and HTqPCR. In detail, the RT‐qPCR cycle threshold, Ct, that was defined from the run as ‘Undetermined’, was assigned a value of ‘Ct = 40’, and the quantile method was used for normalization. We included in our analysis miRNAs with Ct > 13 and < 33 and that were expressed in more than 65% of samples (in each group): 433 miRNAs were included in our analysis. The differential expression of each miRNA has been evaluated by comparing the two staging groups (N1 versus N0): group N0 (13 samples without nodal metastases) and group N1 (15 samples with nodal metastases). Positive FC values refer to miRNAs with higher expression in N1 than in N0, while negative FC values are associated with the opposite situation. To assess statistical differences in the miRNA expression levels between the two groups, we used Wilcoxon rank sum test with Benjamini–Hochberg correction to account for multiple testing.

### Clinical and survival analysis

Clinicopathologic variables for the 28 patients with sporadic MTC (13 N0M0 and 15 N1M0) were extracted from clinical records and are summarized in supplementary material, Table S1. Progression‐free survival (PFS) was defined as the interval from surgery to structural progression or death; patients in structural or biochemical remission at last follow‐up were censored at that time. To investigate factors associated with nodal metastases (N1 versus N0), logistic regression models were fitted, including age at surgery, T stage (T2–3 versus T1), RET mutation category (RET M918T versus other/WT), and *miR‐145‐3p* expression. Associations between miRNA expression and preoperative serum Ctn were assessed using Spearman's rank correlation for each miRNA, and differences in miRNA levels between patients with N0 and N1 were tested with the Mann–Whitney U test. Given the limited sample size and number of events, Cox proportional hazards models were used only in exploratory univariable PFS analyses, and multivariable survival models were not further pursued because of numerical instability and very wide confidence intervals. Kaplan–Meier curves were generated for PFS according to nodal status (N0 versus N1), dichotomized *miR‐145‐3p* expression (high versus low, using the median value), and a composite four‐miRNA panel score derived from the standardized sum of *miR‐145‐3p*, *miR‐24‐3p*, *miR‐126‐3p*, and *miR‐592* expression. Differences between curves were assessed using the log‐rank test.

### Network analysis

We used Spearman's correlation coefficient to assess the level of co‐expression (i.e. coordination of expression profiles) between miRNA in each of the two staging groups (group N0 and group N1).

The obtained co‐expression networks are undirected weighted graphs $G_{i}\left(V,W\right)$ where i=N0,N1, V is the set of vertices (i.e. miRNAs), and W is the set of weighted edges (i.e. Spearman's correlation coefficient) with the weights $w\in \left[-1;1\right]$. The two co‐expression networks were compared at different levels of investigation. Firstly, we evaluated their community structures (Louvain method [9]) to highlight the clusters of co‐expressed miRNAs characterizing each staging group. Then, we computed the differential co‐expression network to establish which pairs of miRNAs are differentially co‐expressed between the two groups. For this second investigation, we assessed the significance of the difference in correlation values with Fisher's z‐test. Thus, we applied the Fisher‐Z transformation on the correlation coefficients and computed *z*‐scores with the following equation:

(1) $$Z_{\mathrm{ij}}=\frac{z_{\mathrm{ij}}^{N1}-z_{\mathrm{ij}}^{N0}}{\sqrt{\frac{1}{n_{N1}-3}+\frac{1}{n_{N0}-3}}}$$

where $z_{\mathrm{ij}}^{N1}$ and $z_{\mathrm{ij}}^{N0}$ are the Fisher‐Z transformation of, respectively, the correlation between miRNAs i and j in the N1 and N0 cohorts, while $n_{N1}$ and $n_{N0}$ are the number of samples of each group ($n_{N1}=15,\;n_{N0}=13$). A positive/negative z‐score indicates gain/loss of coordination between the expression profiles in the N1 group with respect to the N0 condition. Finally, the z‐scores were converted into *p*‐values using the cumulative distribution function (CDF) of the standard normal distribution: we identified significant co‐expression changes setting the *p* value threshold equal to 0.001, and we applied the sign function σ: $Z\to \left\{-1;+1\right\}$.

The obtained differential network is an undirected signed graph $G\left(V,Z,\sigma \right)$ where V is the set of vertices (i.e. miRNAs), Z is the set of edges, and σ is the sign function. The signed adjacency matrix can be expressed as in Equation (2):

(2) $$A=A^{+}-\left|A^{-}\right|$$

where $A^{+}$ is the binary positive component of the differential network (co‐expression stronger in N1), while $A^{-}$ is the binary negative sub‐network (co‐expression stronger in N0).

To identify the most influential nodes within the network, we extracted the degree centrality index measuring the number of connections a node has. In the signed network, it can be calculated as follows:

(3) $$d_{i}^{+}=\frac{\left(\sum_{j}\left|a_{\mathrm{ij}}\right|+\sum_{j}a_{\mathrm{ij}}\right)}{2}$$

(4) $$d_{i}^{-}=\frac{\left(\sum_{j}\left|a_{\mathrm{ij}}\right|-\sum_{j}a_{\mathrm{ij}}\right)}{2}$$

(5) $$d_{i}=d_{i}^{+}+\;d_{i}^{-}$$

where $d_{i}^{+}$ and $d_{i}^{-}$ represent the positive degree and negative degree of node *i* and thus respectively the number of positive and negative edges in which node *i* is involved.

### miRNA network and enrichment pathway analyses

The STRING database version 12 was queried using the miRNA target genes to determine known associations among target genes and perform functional enrichment analysis [10].

Experimental data, databases, and text mining data were used separately at first and then combined to provide target gene interactions. Enrichment pathway analyses were also performed in STRING using the 59 miRNA validated target genes (supplementary material, Table S2) with the following resources: the Kyoto Encyclopedia of Genes and Genomes (KEGG), Gene Ontology‐Biological Process, Gene Ontology‐Molecular Function, and Reactome pathways. The top 15 pathways for each resource with a false discovery rate (FDR) ≤ 0.05, grouped by similarity ≥ 0.8 and sorted by signal, were selected for visualization, and images were generated using STRING.

### Cell culture and miRNA transfection

Functional studies were performed on the MTC‐derived TT cell line, which harbors the C634W RET mutation and produces calcitonin [11]. TT cells were obtained from American Type Culture Collection (ATCC, Manassas, VA, USA) and grown in Ham's F‐12K (Kaighn's) Medium (Cat. 21127022; Gibco, Thermo Fisher Scientific) supplemented with 10% (v/v) fetal bovine serum (FBS, Cat. A5256701; Gibco), and 1% (v/v) penicillin–streptomycin. Cells were cultured in a humidified atmosphere of 95% air and 5% CO_2_ at 37 °C. To evaluate the biological role of key miRNAs, miRCURY LNA miRNA Inhibitors were used (Cat. 339121; Qiagen): hsa‐miR‐145‐3p (GeneGlobe Id: YI04103052‐ADA), hsa‐miR‐126‐3p (GeneGlobe Id: YI04109148‐ADA), hsa‐miR‐24‐3p (YI04101706‐ADA), hsa‐miR‐592 (GeneGlobe Id: YI04100911‐ADA), Negative control A (Qiagen, Cat. No. 339126, GeneGlobe Id: YI00199006‐ADA). miRNA inhibitors were transfected as a pool containing 10 nm each using Hiperfect (Cat. No./ID: 301705, Qiagen) twice, and results were evaluated 48 h after the second transfection. Cell proliferation was evaluated by using CellTiter 96 Aqueous One Solution Cell Proliferation Assay (MTS) (Cat. G3580, Promega, Madison, WI, USA). Cell death was assessed by trypan blue exclusion.

### Western blotting

Cells were lysed in 50 mm Tris–HCl pH 7.8, 10% SDS, 10% glycerol, 10 mm EDTA pH 8.0, 100 mm NaF, 10 mm Na_2_P_2_O_7_, 6 M urea, and protease inhibitors as described in the manuscript by Trocchianesi *et al* 2023 [12]. Lysates were separated on 8% acrylamide gel and immunoblotted using standard procedures. Antibodies: anti‐p‐ERK1/2 (#sc‐101,761; Santa Cruz Biotechnology, Dallas, TX, USA), anti‐ERK1/2 (#137F5; Cell Signaling Technology Inc., Danvers, MA, USA), and anti‐vinculin (7F9) (#sc‐ 73614; Santa Cruz Biotechnology). HRP‐conjugated secondary antisera (Santa Cruz Biotechnology), followed by enhanced chemiluminescence (ECL Amersham, Cytiva/GE Healthcare UK Limited, Amersham, UK), were used. Chemiluminescence was recorded using Chemidoc (Bio‐Rad Laboratories Inc., Hercules, CA, USA), and band intensity was assessed using ImageJ (US National Institutes of Health, Bethesda, MD, USA).

### RNA extraction and quantitative real‐time PCR

RNA was isolated from cells using TriZol (Cat. 5596026, Invitrogen) and retro‐transcribed using the High‐Capacity cDNA Reverse Transcription kit (Cat. 4368814, Life Technologies). RNA expression was analyzed using the QuantStudio 3 Real‐Time PCR System (Life Technologies) and SensiFAST Probe Lo‐ROX (Bioline, Memphis, TN, USA), where required. mRNA quantification was expressed in arbitrary units, as the ratio of the sample quantity to the calibrator or to the mean values of control samples. For gene expression analysis, the following gene expression assays were used: *CCND1* (Hs00765553_m1), *MYCN* (Hs00232074_m1) and NF‐kB *p65* (Hs01042014_m1), *HES1* (Hs00172878_m1), *HES5* (Hs01387463_g1), *TWIST1* (Hs00361186_m1), and *TWIST2* (Hs00382379_m1). Values were normalized to two endogenous controls: *GAPDH* (Hs99999905_m1) and *β‐ACTIN* (Hs99999903_m1).

### Statistical analyses

For cell proliferation, cell death, and protein expression, data are presented as mean ± SD of at least three independent experiments. Statistical analyses were performed by the Student's *t*‐test using the GraphPad Prism Software v.9.0 (GraphPad Software, San Diego, CA, USA; https://www.graphpad.com, last accessed 10 May 2026). *p* values less than 0.05 were considered statistically significant.

## Results

### Network analysis of MTC with or without nodal metastasis

Lymph‐nodal metastasis can be an early event in MTC pathogenesis; therefore, we obtained microRNA profiles and performed co‐expression network analysis in a cohort of 28 patients with MTC. Biological specimens were collected at the time of thyroidectomy from patients with (N1) or without (N0) nodal metastasis at diagnosis (supplementary material, Table S1). We determined the community structure of the two co‐expression networks, identifying 433 miRNAs (Figure 1A–D). In N0 (Figure 1A left plot and Figure 1C), the network shows two main clusters composed of 217 and 210 miRNAs, fuchsia and turquoise, respectively. These two clusters showed a high number of positive within‐cluster connections and were strongly anticorrelated with each other. Conversely, in the N1 group (Figure 1A right plot and Figure 1D), we observed a re‐organization of miRNAs, resulting in the formation of three new clusters: blue, orange, and yellow blocks. The blue community was mainly composed of miRNA belonging to the fuchsia community in N0, the orange one of miRNAs belonging to the turquoise community in N0, while some miRNAs from the fuchsia and turquoise communities in N0 converged in the yellow block (Figure 1B). In general, we observed that co‐expression changes of miRNAs belonging to clusters in N0 lead them to create a new cluster in N1. These results suggest a profound reorganization of miRNA networks during the progression from the N0 to N1 condition.

**Figure 1:**
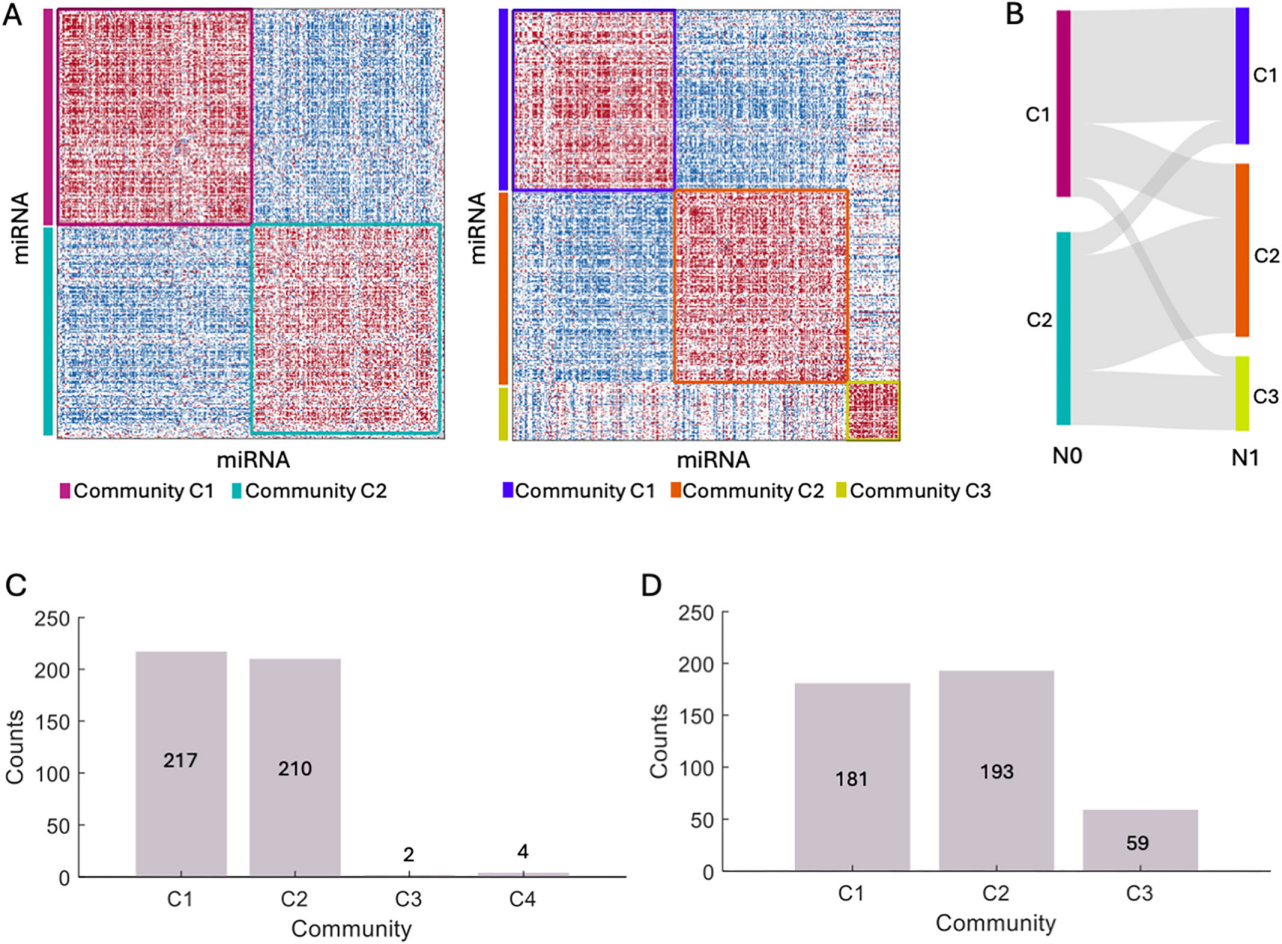
Co‐expression networks of N0 and N1 staging groups. (A) Adjacency matrices showing the community structure of the two networks: N0 on the left and N1 on the right; the blue scale indicates negative correlation coefficients, while the red scale indicates positive correlation coefficients. (B) The Sankey diagram shows the reorganization of the network in N1 with respect to N0 condition. (C) Bar chart of community size in N0. (D) Bar chart of community size in N1.

### Network analysis highlighted the central role of *miR‐145‐3p*

To better comprehend the miRNA reorganization and characterize the miRNA interactions between the N0 and N1 subgroups, we performed differential co‐expression analysis (Figure 2A). This approach generated a network in which each node represents an individual miRNA, while the edge between them indicates changes in miRNA‐miRNA co‐expression between the two conditions N0 and N1. Specifically, blue edges represent interactions that are stronger in the N0 group, while the red indicate the ones that are stronger in the N1 group. The analysis revealed that among the 433 miRNAs analyzed, 189 appeared as isolated nodes, suggesting that they are not involved in differential co‐expression changes between the N0 and N1 staging. Of note, 244 miRNAs were involved in the largest connected component of the differential network (Figure 2A left side). The distributions of the total degree, positive degree, and negative degree (Figure 2A right side) revealed that most miRNAs have few connections and that more connections get lost from N0 to N1 than new connections in N1 that were not present in N0. Interestingly, it emerges that *miR‐145‐3p* was involved in 29 significant statistical co‐expression changes, where 19 represent co‐expression patterns stronger in N0 and the remaining 10 co‐expression links stronger in N1 (Figure 2B). *miR‐145‐3p* resulted in being the central node of the co‐expression network hub, playing a key role in distinguishing the two staging groups. We therefore investigated its expression level, along with its closest neighbors (CN), miRNAs directly linked to *miR‐145‐3p*. Figure 2B shows the differential network centered around *miR‐145‐3p*: node colors represent the group where they are present with higher expression (blue for N0 and red for N1), the node size reflects the magnitude of miRNA differential expression, while the edge color represents the statistical changes of the miRNA‐miRNA co‐expression (blue for a loss of coordination in N1 with respect to N0 and red for co‐expression stronger in N1 than in N0). Focusing on N0, we observe higher expression of *miR‐145‐3p* along with 16 CN co‐expressed miRNAs. Moreover, we report the expression levels of *miR‐145‐3p* and its closest neighbors in N1 compared with the N0 group (Figure 2C and supplementary material, Table S3). Since *miR‐145‐3p* expression was higher in the N0 group, we focused on the network constituted by its N0 closest neighbors (N0CN). This network is silenced/weakened in N1 compared with N0, suggesting that the molecular mechanisms underlying this behavior might be lost with MTC progression.

**Figure 2:**
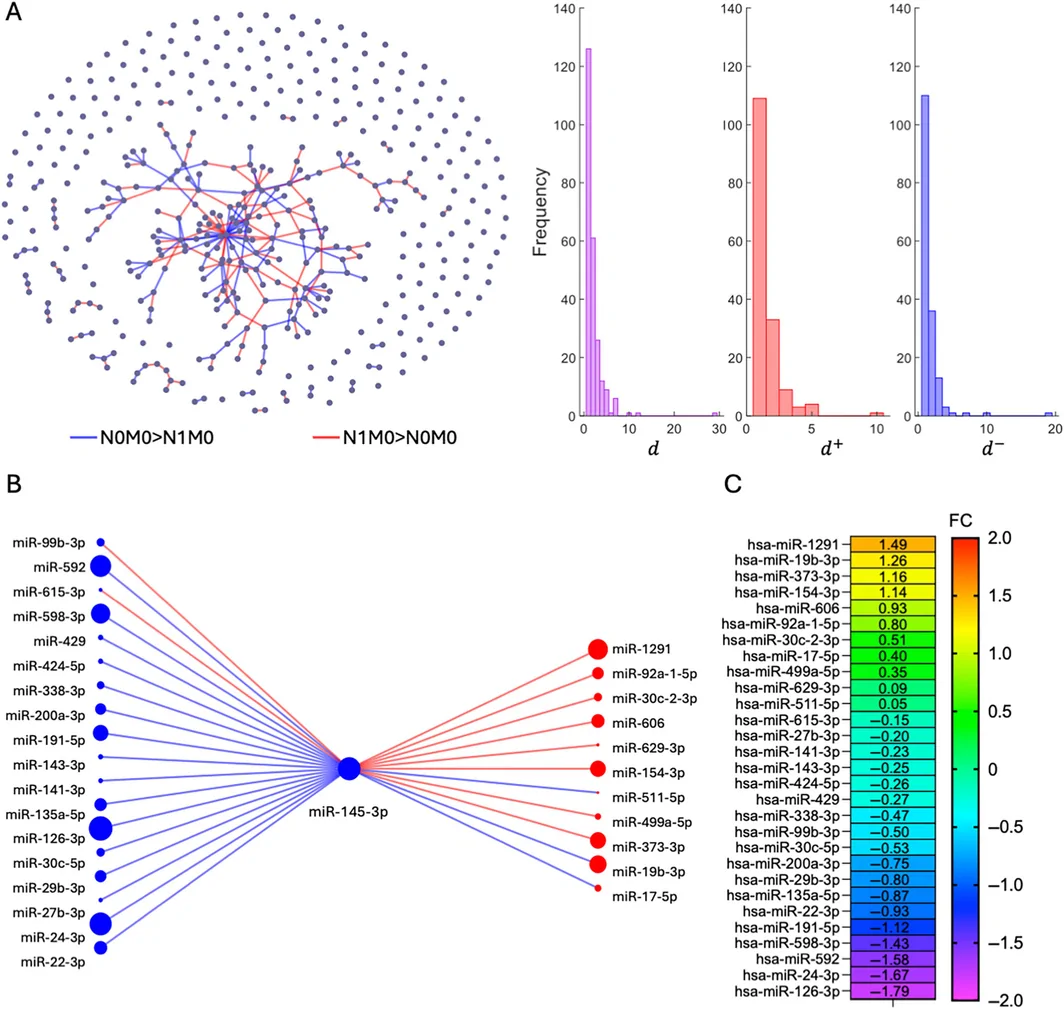
miRNA differential network analysis between N0 and N1 staging groups. (A) On the right: differential co‐expression network with nodes representing miRNAs and links informing about statistical co‐expression changes between N0 and N1 staging groups: blue links inform about stronger co‐expression in N0, while red links about stronger co‐expression in N1. On the left, the distribution of the total degree d (in violet), the positive degree $d^{+}$ (in red), and the negative degree $d^{-}$ (in blue). The number of miRNAs with *d* = 0 is not shown. (B) Sub‐graph of the network hub: the first neighbors of *miR‐145‐3p* are displayed in separate layers to highlight the agreement between change in co‐expression and change in expression level between N0 and N1: link color code is the same as in panel A, while node color codes for negative fold‐change (FC) (blue) or positive FC (red), and node size is proportional to the FC absolute value. (C) The table reports FC values of miRNAs directly linked to hsa‐miR‐145‐3p.

### Enriched pathways analysis of genes targeted by *miR‐145‐3p* and its N0CN

Since the N0 network is active in the context of a localized disease, we hypothesized that it may govern the non‐metastatic phenotype. We therefore investigated the molecular pathways potentially regulated by these miRNAs through MIENTURNET enrichment using miRTarBase, thus identifying 59 validated target genes (supplementary material, Table S2) with significant FDR values. To better understand the intricate regulatory system of the N0 landscape, we performed a protein–protein interactions (PPI) analysis on the target genes using different data sources, including experimental data, databases, and text mining. This approach identified PPI information for 40 genes (Figure 3A). The combination of the three data sources enabled the identification of an interaction network that includes several genes involved in cancer development and maintenance, such as *MYC*, *PTEN*, *BCL2*, *PIK3CA*, *AKT*, and *MAPK7*, among others.

**Figure 3:**
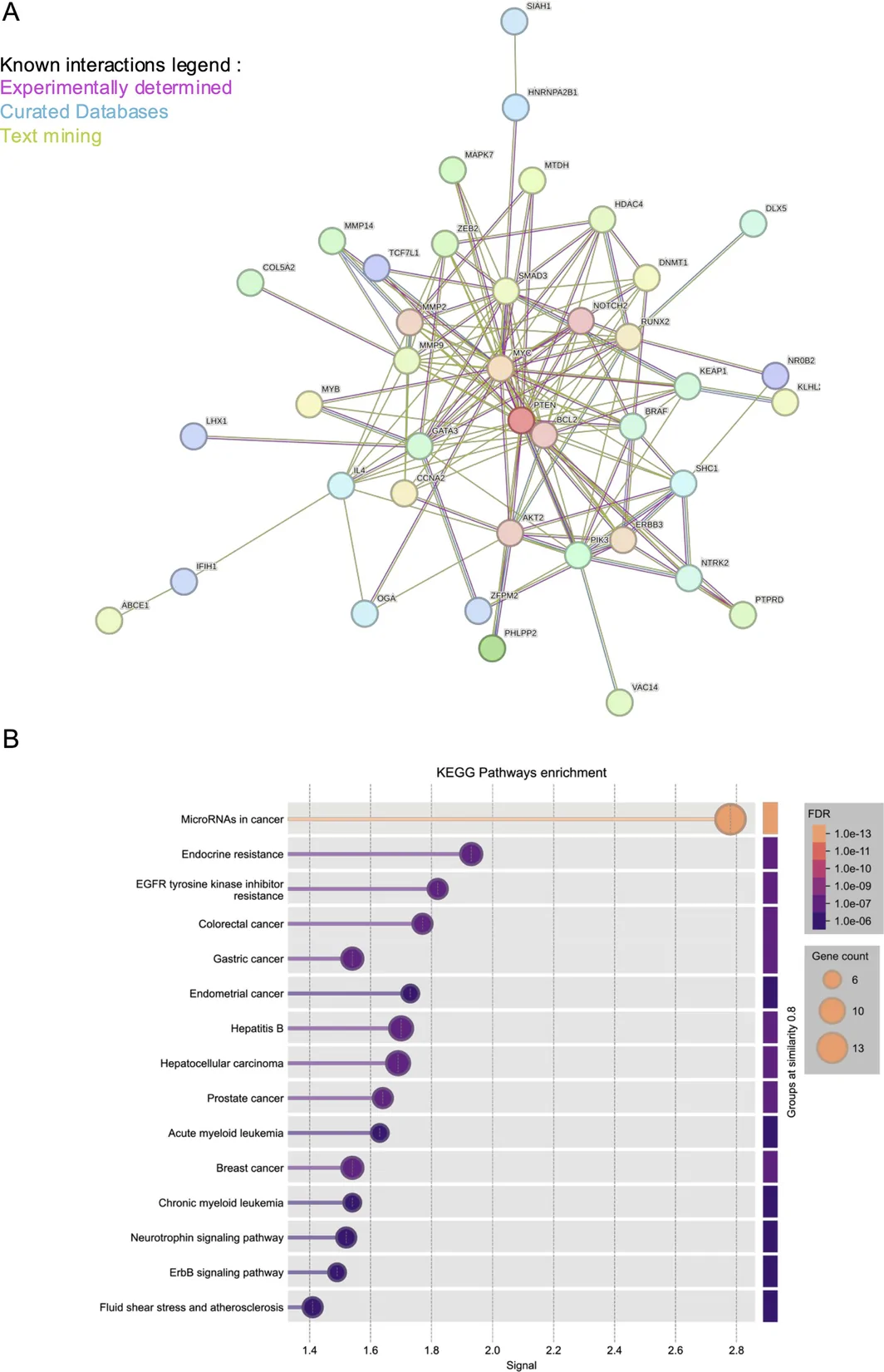
miRNA target protein–protein interaction networks and Kyoto Encyclopedia of Genes and Genomes (KEGG) enrichment pathway analyses. (A) Protein–protein interaction network of 40 miRNA target genes using experimentally determined data (purple lines), curated databases (turquoise lines), and text mining (green lines) resources. (B) Top 15 enriched pathways with FDR≤0.05 were selected using KEGG. Enriched pathways are grouped by similarity ≥ 0.8 and sorted by signal.

We further investigated the N0 network target genes through enrichment pathway analysis to shed light on signaling pathways that characterize the non‐metastatic processes. To pursue this aim we used three different databases: KEGG to obtain information about canonical metabolic or signaling pathways in which these genes are involved; Gene Ontology, which provides hierarchical and standardized vocabulary describing gene functions in terms of ‘Biological Processes’, thus identifying which biological processes participate and ‘Molecular Functions’, highlighting their specific biochemical activities; and ‘Reactome’, which contains detailed molecular reactions, including physical and temporal interactions. Of note, it emerged that the top three enriched pathways for the KEGG database were miRNAs involved in cancer, endocrine resistance, and EGFR tyrosine kinase inhibitor resistance (Figure 3B). ‘Biological Process' from Gene Ontology revealed negative regulation of apoptotic process, cell death, and differentiation (Figure 4A), while ‘Molecular Function’ enrichment indicated that the target genes were involved in protein binding and DNA binding activities (Figure 4B). For instance, analysis conducted on the ‘Reactome’ database highlighted an enrichment of signaling pathways related to receptor tyrosine kinases (RTKs), immune system, cytokines, and estrogen pathways (Figure 4C). Multiple enrichment pathway analyses pointed out the key role of the miRNA network characterizing the N0 condition and of the 59 targeted genes (Figure 3B and Figure 4) in regulating the cancer‐related processes. Interestingly, in MTC biology, RET or RAS hyperactivation triggers the activation of RAS/RAF/MEK/ERK and PI3K/AKT pathways, shaping proliferation, survival, and therapeutic response [13]. Based on these results, we chose to focus on the RTK/RAS/RAF/MEK/ERK axis in MTC and evaluate the biological role of *miR‐145‐3p* and its associated N0CN miRNAs.

**Figure 4:**
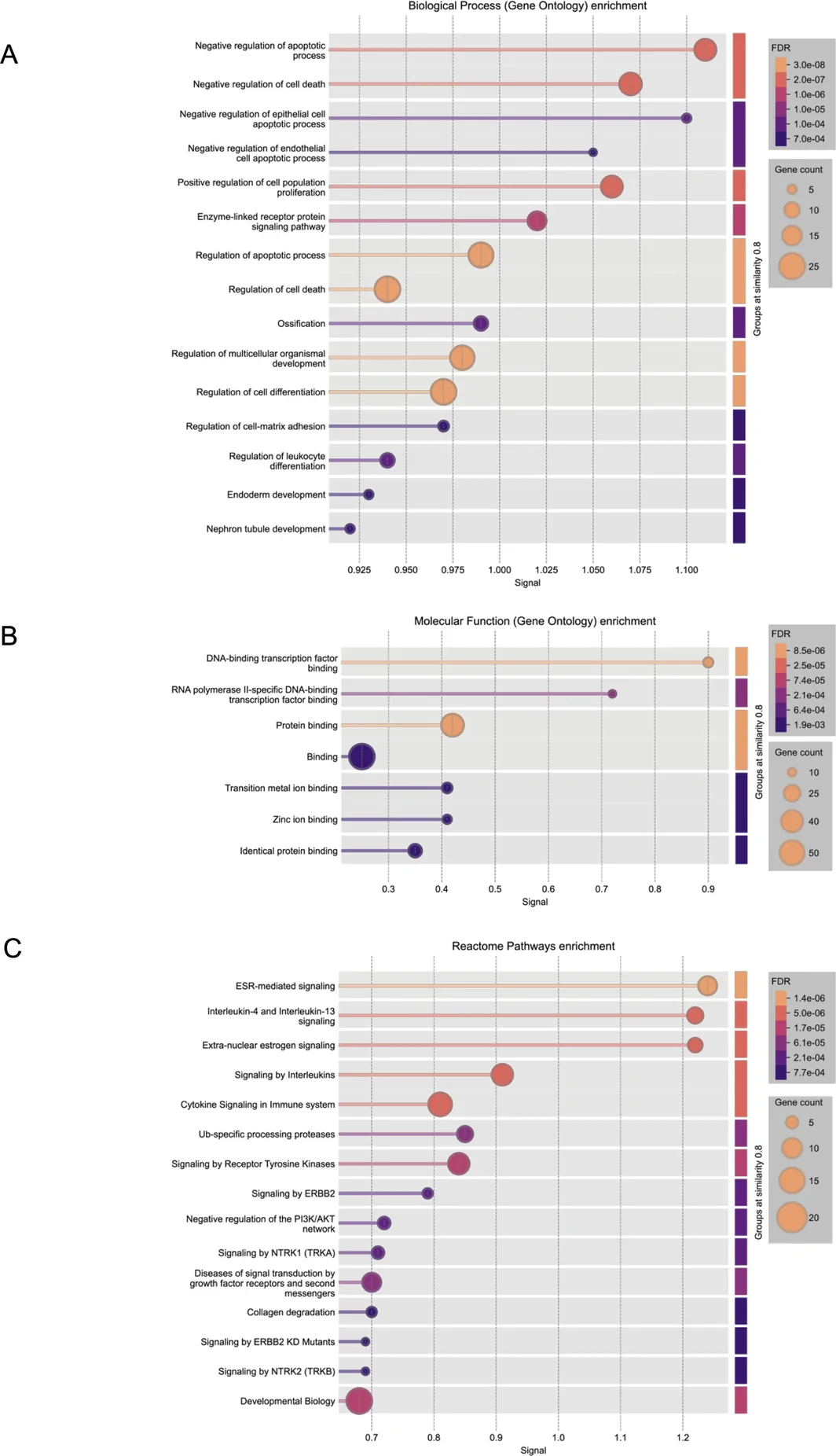
Gene Ontology and Reactome enrichment pathway analyses of miRNA target genes. Top 15 enriched pathways with FDR ≤ 0.05 were selected using (A) Biological Process (Gene Ontology), (B) Molecular Function (Gene Ontology), and (C) Reactome resources. Enriched pathways are grouped by similarity ≥ 0.8 and sorted by signal.

### Clinical and functional assessment of a four‐miRNA network associated with N0 MTC

We focused on a subset of four miRNAs (*miR‐145‐3p*, *miR‐126‐3p*, *miR‐24‐3p*, and *miR‐592*) selected from the N0‐correlated network based on high fold change in N0 samples, strong positive correlation with *miR‐145‐3p*, and marked differential expression between N0 and N1 groups (supplementary material, Table S3). To explore the potential clinical relevance of these miRNA signatures, we first assessed their association with nodal status, pre‐operative Ctn levels, and PFS in our cohort of 28 patients. In multivariable logistic regression including age, T stage, and RET M918T mutation status, all clinical variables were significantly associated with N1 status (supplementary material, Table S4, model 1). However, *miR‐145‐3p* was not independently associated with nodal involvement when added to the model (adjusted OR 1.29, 95% CI 0.96 to 1.95, *p* = 0.14; supplementary material, Table S4, model 2). Consistently, while the N1 group showed worse PFS compared with the N0 group (*p* = 0.042; supplementary material, Figure S1A), neither *miR‐145‐3p* nor the 4‐miRNA panel significantly stratified clinical outcomes (supplementary material, Figure S1B,C). Moreover, no significant associations emerged between the miRNAs and nodal status or pre‐operative Ctn levels (supplementary material, Table S5). These analyses did not support significant clinical associations, although the limited sample size and low event rate substantially reduce the statistical power of these correlations. To functionally validate the biological role of this miRNA network, we performed combined inhibition of the four miRNAs in MTC‐derived TT cells and assessed proliferation and cell death. Our results showed that inhibition of these miRNAs significantly promoted proliferation while showing a trend of cell death attenuation (Figure 5A). To evaluate the invasive ability of TT cells following the inhibition of *miR‐145‐3p* and its N0CN we performed a wound‐healing assay. We observed that the inhibition led to faster wound closure compared with the control condition, although the difference did not reach statistical significance (supplementary material, Figure S2A).

**Figure 5:**
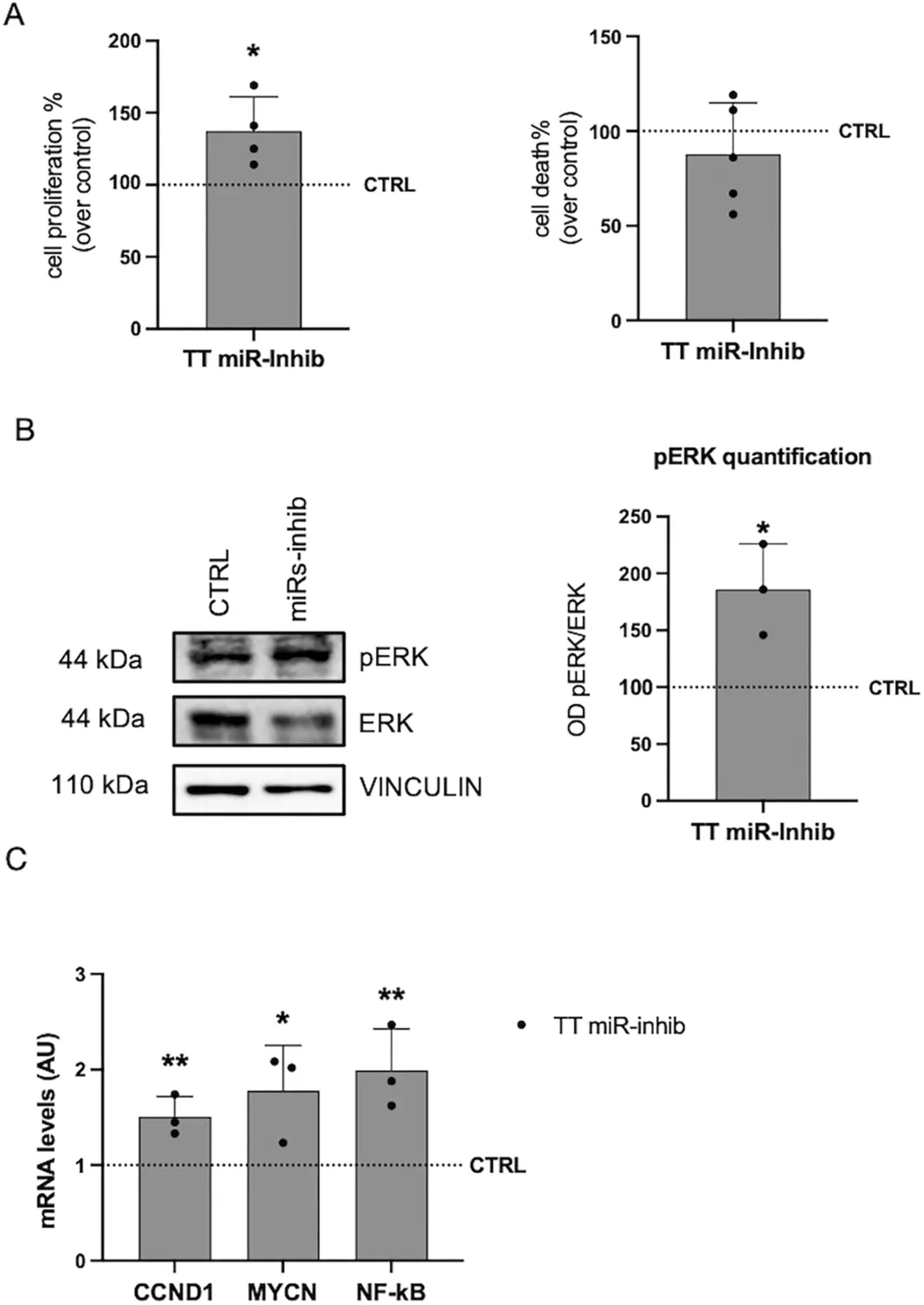
*miR‐145‐3p* network controls medullary thyroid carcinoma (MTC) cell cancer features. (A) Cell proliferation and cell death in TT after inhibition of *miR‐145‐3p*, *miR‐126‐3p*, *miR‐24‐3p*, and *miR‐592*. Bar plots are representative of three independent experiments. Paired *t*‐test was performed, *p < 0.05. (B) Representative blot images and histogram of densitometry analysis of pERK/ERK ratio after *miRNA* inhibition as described for panel A. Bar plots are representative of three independent experiments. Paired *t*‐test was performed, *p < 0.05. (C) Gene expression of Cyclin D1 (*CCND1*), *MYCN*, and *NF‐kB* in TT cells after miRNA inhibition as described for panel A; dashed line: control. Paired *t*‐test was performed, *p < 0.05, **p < 0.01.

In addition, we evaluated if the inhibition of the four miRNAs could impact on MAPK and AKT pathways, critical signaling pathways frequently hyperactivated in MTC [12]. In this context, western blotting analysis revealed an increase in ERK phosphorylation levels (Figure 5B), while no modulation was detected for phospho‐AKT (pAKT) (supplementary material, Figure S2B). To further investigate the role of *miR‐145‐3p* and its N0CN, we performed real‐time quantitative PCR analysis of *MYCN*, *CCND1*, *NF‐κB*, *TWIST1*, *TWIST2*, *HES1*, and *HES5*. The rationale for these analyses was as follows: *MYCN* was evaluated to assess the activation of the MAPK signaling pathway [14] and because it is a validated marker of *miR145‐3p*; *CCND1* was analyzed because it is considered a marker of advanced MTC [15]; NF‐κB was included due to its established role in MTC progression and metastasis [16]; and *TWIST1, TWIST2*, *HES1*, and *HES5* were selected as well‐known epithelial–mesenchymal transition (EMT) markers in several tumor types [17, 18, 19]. Interestingly, we observed a statistically significant increase in *MYCN*, *CCND1*, and *NF‐κB* mRNA levels following the inhibition of *miR‐145‐3p* and its N0CN (Figure 5C). *TWIST1*, *TWIST2*, *HES1*, and *HES5* showed an increasing trend, although the differences were not statistically significant (supplementary material, Figure S2C). Altogether, these findings indicate that disruption of the miRNA network characterizing N0 MTC tissues promotes a more aggressive cellular phenotype, supporting a functional role for this network in restraining tumor progression.

## Discussion

MTC is a rare neuroendocrine tumor [20] whose prognosis worsens significantly with lymph node metastases [21]. Understanding molecular mechanisms underlying the N0–N1 transition is therefore critical for therapeutic management. In this context, miRNAs are key players in MTC pathogenesis [22] and their dysregulation reflects specific oncogenic processes and supports their role as potential biomarkers [23]. For example, upregulation of *miR‐183‐5p* and *miR‐375‐5p* has been linked to proliferation, apoptosis resistance, and metastatic dissemination via PI3K/AKT pathways, while reduced *miR‐200c* expression is associated with EMT through ZEB1/ZEB2 and E‐cadherin modulation [24]. Because miRNAs typically act collectively – each targeting multiple mRNAs and converging on shared signaling pathways—the transcriptional program of cancer cells is orchestrated by complex regulatory networks in which the epigenetic layer plays a central role [25]. We therefore applied network analysis to identify miRNA networks associated with N0 and N1 conditions, focusing on co‐expression patterns rather than differential expression alone. Our analysis of 28 patients with MTC identified several miRNAs involved in the transition from N0 to N1 stage. While the N0 network displayed two main clusters with opposite expression patterns, the N1 stage exhibited a reorganized architecture with three distinct clusters, indicating increased complexity during progression. By analyzing the differential co‐expression network, *miR‐145‐3p* emerged as a critical hub bridging the N0 and N1 networks.

*miR‐145‐3p* is recognized as a tumor suppressor frequently downregulated in multiple cancers, including lung adenocarcinoma and head and neck squamous cell carcinoma, where it targets the oncodriver gene *MYO1B* [26, 27]. Although direct data on *miR‐145‐3p* in MTC are limited, *miR‐145* family members are consistently under expressed in thyroid malignancies. In papillary thyroid carcinoma (PTC), overexpression of *miR‐145* suppresses tumor progression by targeting PI3K/AKT‐ and N‐cadherin‐mediated pathways, thereby inhibiting proliferation, invasion, and EMT [28, 29]. These suppressive mechanisms may be relevant to MTC pathogenesis and warrant further investigation, given the central role of PI3K/AKT and MAPK signaling in both PTC and MTC [29]. Enrichment analysis of validated targets of *miR‐145‐3p* and its N0‐correlated network (N0CN) revealed over‐representation of pathways linked to MTC biology, including microRNAs in cancer, endocrine resistance, and multiple cancer‐related signaling cascades, as well as biological processes such as negative regulation of apoptosis and cell differentiation. Notably, the network included high‐connectivity oncogenic hubs such as MYC, PTEN, BCL2, PIK3CA, AKT1, and MAPK7. Reactome pathway annotations further emphasized RTK and immune system signaling, both critical to MTC tumorigenesis. Dysregulation of RTK signaling in MTC most often results from activating mutations in RET or, in RET wild‐type cases, mutations in RAS, both converging on the RAS/RAF/MEK/ERK cascade [22]. The inclusion of PIK3CA, AKT1, and MAPK7 among *miR‐145‐3p*'s potential targets suggests that its downregulation contributes to unchecked MAPK and PI3K/AKT pathway activity in MTC. To validate these findings functionally, we inhibited *miR‐145‐3p* along with three top‐ranking N0CN miRNAs (*miR‐126‐3p, miR‐24‐3p*, and *miR‐592*) in TT cells. Their concurrent inhibition significantly increased cell proliferation, showed a trend toward reduced cell death, and enhanced ERK phosphorylation, consistent with MAPK pathway activation. Moreover, inhibition resulted in increased mRNA levels of *MYCN*, *CCND1*, and *NF‐κB*, well‐established markers of MTC progression. Taken together, these findings support a model in which *miR‐145‐3p* and its N0CN act as a negative regulator of RTK/MAPK/PI3K signaling in MTC, and their reduced expression may facilitate tumor progression by enabling simultaneous activation of multiple growth‐promoting pathways (Figure 6). By leveraging network analysis to dissect the regulatory landscape of MTC, our study moves beyond single‐miRNA approaches and highlights differential miRNA networks between localized and early metastatic disease. This system‐level perspective identifies not only individual miRNAs of interest but also broader regulatory circuits that could serve as combinatorial therapeutic targets or prognostic signatures.

**Figure 6:**
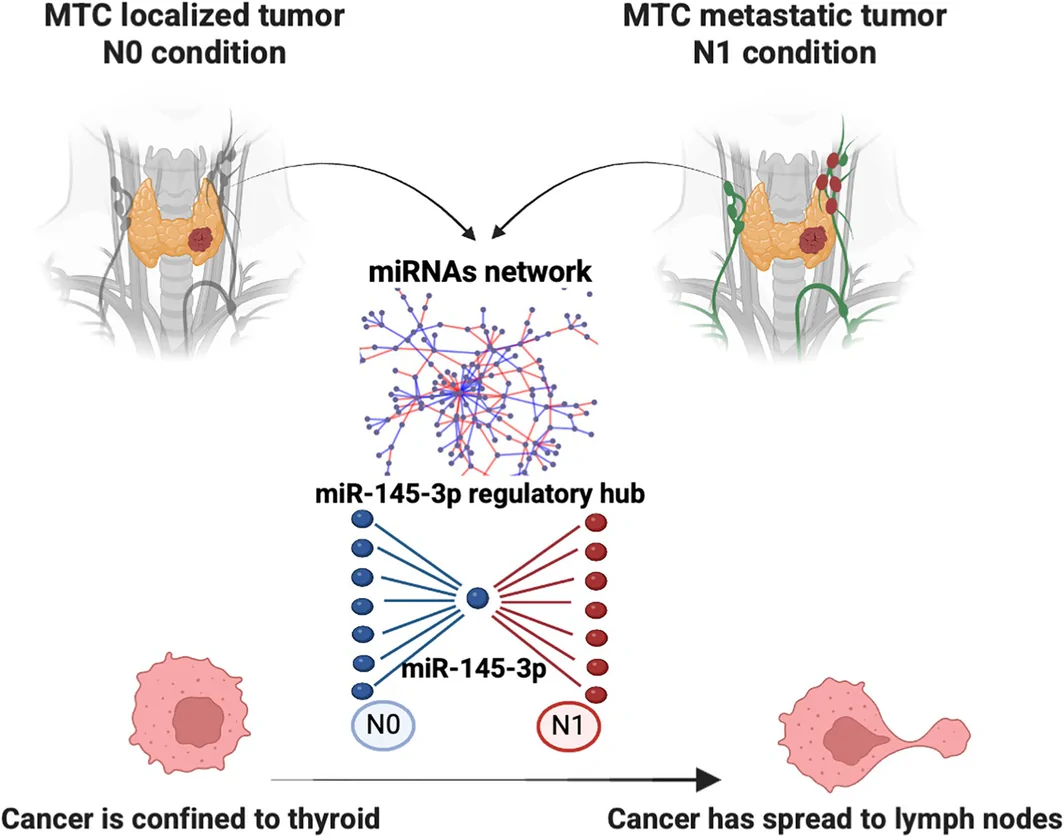
*miR‐145‐3p*‐centered miRNA network rewiring during the N0 to N1 transition in medullary thyroid carcinoma (MTC). Network analysis on N0 and N1 MTC samples revealed the presence of a regulatory hub with *miR‐145‐3p* as the central node governing the N0 to N1 transition. Created with Biorender.com.

MTC is a rare type of thyroid cancer with fewer than 0.3 of new diagnoses per 100,000 in women and 0.2 per 100,000 in men per year in Europe [30]. This low incidence poses significant challenges in biomedical research, limiting large‐scale studies. Our study, based on a relatively small cohort of patients and a single commercially available MTC cell line, limits the extent to which our findings can be generalized to the broader MTC population. We are aware that the restricted number of cases and the use of one *in vitro* model do not fully capture the genetic, epigenetic, and phenotypic heterogeneity that characterizes MTC in clinical practice. A formal *a priori* power analysis was not performed, as patient recruitment was inherently limited by disease rarity. The sample size was therefore determined by feasibility and consecutive case availability rather than predefined statistical assumptions. Nonetheless, the network analytical approach allowed us to obtain meaningful results from a small cohort of patients and may be particularly useful for the study of rare tumors, where the combined action of multiple molecules can still be highlighted despite the limited case availability.

## Authors contributions statement

AC, MP, ZMB, AP and EF contributed to the conceptualization of the study, methodology, writing of the original draft of the manuscript and data curation. AC, MP, ZMB, CA, ES, ST, SaC, MC, CS, FG, TMA, MB, GC, AV, RE, RC, CR, MGC, SiC, VM, LP, LF and CD contributed to formal analysis, investigation, and validation. AV, RE, RC, CR, MGC, SiC, LF, AP and EF participated in the review and editing of the manuscript. EF acquired funding for the study. All authors read and approved the final manuscript.

## Supporting information


**Figure S1.** Kaplan–Meier analysis of progression‐free survival in patients with medullary thyroid carcinoma (MTC) stratified by nodal status, *miR‐145‐3p* expression, and combined miRNA signature
**Figure S2.** *miR‐145‐3p* network controls medullary thyroid carcinoma (MTC) cells cancer features

**Table S1.** Medullary thyroid carcinoma (MTC) patients' clinical features
**Table S2.** List of hsa‐*miR‐145‐3p* and 16 N0CN miRNAs along with their target genes
**Table S3.** Features of miRNAs connected to hsa‐*miR‐145‐3p* in N0
**Table S4.** Multivariate logistic regression analysis for nodal metastases (N1 versus N0)
**Table S5.** Association of miRNAs with nodal status and pre‐operative calcitonin levels

## Data Availability

The datasets used and/or analyzed during the current study are available from the corresponding author on reasonable request.
