# Supplementary material for: Network based approach identifies miR ‐145‐3p as a central regulatory hub associated to the progression from localized to metastatic medullary thyroid carcinoma
Source: J Pathol. 2026 Sep 9;270(4):468–80. doi: 10.1002/path.70116 (PMC13728816; doi:10.1002/path.70116)
Supplement: Supplementary file 1 [file PATH-270-468-s002.docx]

**Network based approach identifies *miR-145-3p* as a central regulatory hub driving the shift from localized to metastatic medullary thyroid carcinoma**

A Citarella, M Petti *et al J Pathol*. <https://doi.org/10.1002/path.70116>

**Supplementary Figures S1, S2**

**Supplementary Tables S1–S5** (provided as a separate Excel file)

**Figure S1. Kaplan–Meier analysis of progression-free survival in patients with medullary thyroid carcinoma (MTC) stratified by nodal status, *miR-145-3p* expression, and combined miRNA signature.** Progression of disease (PoD) or death was considered an event, whereas patients whose condition was in remission at their last follow-up were censored. **(A)** Progression-free survival according to lymph-node metastasis status. Patients with N0 disease ($n=12$) had significantly higher progression-free survival than patients with N1 disease ($n=13$; $p=0.042$, log-rank test). No progression or death events were observed in the N0 group during the available follow-up, and the Kaplan–Meier estimate remained at 100%. In the N1 group, estimated progression-free survival was 61.5% (95% CI 40.0% to 94.6%) at 40 months and 30.8% (95% CI 12.4% to 76.3%) at 96 months. **(B)** Progression-free survival according to miR-145-3p expression. Patients were dichotomized at the median expression value into low-expression (< median; $n=12$) and high-expression ($\geq$ median; $n=13$) groups. No statistically significant difference in progression-free survival was observed between the groups ($p=0.44$, log-rank test). At 48 months, estimated progression-free survival was 65.6% (95% CI 35.2% to 100.0%) in the low-expression group and 63.5% (95% CI 40.0% to 100.0%) in the high-expression group. **(C)** Progression-free survival according to a four-miRNA panel score comprising *miR-24-3p*, *miR-126-3p*, *miR-592*, and *miR-145-3p*. The panel score was calculated as the unweighted sum of the standardized expression values of the four miRNAs. Patients were dichotomized at the median score into low-panel (< median; $n=12$) and high-panel ($\geq$ median; $n=13$) groups. No statistically significant difference in progression-free survival was observed between the groups ($p=0.65$, log-rank test). At 48 months, estimated progression-free survival was 51.4% (95% CI 21.3% to 100.0%) in the low-panel group and 74.0% (95% CI 52.2% to 100.0%) in the high-panel group. Time is expressed in months, and the numbers at risk are shown below each survival curve at the indicated time points. Shaded bands represent pointwise 95% confidence intervals based on Greenwood standard errors, and plus signs indicate censored observations.

**Figure S2. *miR-145-3p* network controls medullary thyroid carcinoma (MTC) cell cancer features.** (A) Brightfield images of wound healing assay in TT cells after the inhibition of *miR-145-3p*, *miR-126-3p*, *miR-24-3p*, and *miR-592*. Bar plots are representative of three independent experiments. Paired *t*-test was performed, **p* < 0.05. (B) Representative blot images and histogram of densitometry analysis of pAKT/AKT ratio after miRNA inhibition as described for panel A. Bar plots are representative of three independent experiments. Paired *t*-test was performed, **p* < 0.05. (C) Gene expression of *HES1*, *HES5*, *TWIST1*, and *TWIST2* in TT cells after the miRNA inhibition as described for panel A; dashed line: control. Paired *t*-test was performed, **p* < 0.05, ***p* < 0.01.
